# Change in the site density and surface acidity of clay minerals by acid or alkali spills and its effect on pH buffering capacity

**DOI:** 10.1038/s41598-019-46175-y

**Published:** 2019-07-08

**Authors:** Inhyeong Jeon, Kyoungphile Nam

**Affiliations:** Department of Civil and Environmental Engineering, Seoul National University, Seoul, 08826 Republic of Korea

**Keywords:** Geochemistry, Environmental monitoring

## Abstract

Changes in the site density and surface acidity constants (i.e. pKa_1_ and pKa_2_) of kaolinite and montmorillonite were determined after acid or alkali spills, and pH buffering capacity was evaluated as a parameter of soil function change. Surface complexation modeling with potentiometric titrations and Fourier-transform infrared spectroscopy showed that acid or alkali spills did not significantly change the surface properties of kaolinite. In montmorillonite, however, acid spills decreased the basal site density from 832 to 737 mmol kg^−1^ by dissolving substituted octahedral cations and decreased pKa_2_ from 7.32 to 5.42 by dissolving SiOH. In response to alkali spills, the basal site density increased to 925 mmol kg^−1^, and the edge site density increased from 84.8 to 253 mmol kg^−1^ due to AlOH and SiOH formation; thus, pKa_2_ decreased to 6.78. The pH buffering capacity of acid- or alkali-spilled kaolinite at pH 6 did not significantly change, while that of acid- or alkali-spilled montmorillonite increased from 30.3 to 35.9 and 56.0 mmol kg^−1^, respectively. Our results indicate that these spills greatly altered the surface properties of montmorillonite, but unexpectedly, increased the pH buffering capacity of montmorillonite.

## Introduction

As the chemical industry develops, chemical accidents occur annually, and of them, acid or alkali spills are of great concern because of their high frequency and hazard^1^. According to the Chemistry Safety Clearing-house database^2^, acid or alkali spills accounted for 46% of the chemical accidents in South Korea^3,4^. If acid or alkali spills onto soil, most of the physicochemical properties of soil are altered, such as pH, organic matter content, base saturation, exchangeable cations, and surface area^5–9^. Although neutralizers have been poured onto acid- or alkali-spilled soils for pH recovery^10,11^, neutralization cannot recover nonreversible dissolution of organic matter or clay minerals and the change in cation exchange capacity (CEC) caused by the structural deterioration^12^. Changes in these properties will alter soil functions, particularly pH buffering capacity. Because pH buffering capacity is related to soil productivity and the water quality of stream water near soil^13^, studies of the change in pH buffering capacity of acid- or alkali-spilled soil after neutralization are needed.

For this purpose, the effects of acid or alkali spills on organic matter and clay minerals, the soil constituents that determine the pH buffering capacity of soils, should be analyzed. In the case of organic matter, acid or alkali spills desorb or dissolve it^14–16^; thus, the pH buffering capacity decreases. However, the effect of these spills on clay minerals is unclear. Little attention has been paid on changes in clay minerals’ properties due to these spills. Only a few studies revealed that an acid or alkali treatment at room temperature over two weeks not only altered a crystal structure of clay minerals but also increased surface area^8^. Instead, many studies have focused on an acid or alkali activation treating clay minerals with high concentration of acid or alkali, and its effect on clay minerals’ surface area, porosity and surface acidity^17–19^. Also, there have been several studies on soil acidification phenomenon, and they revealed that the long-term acidification led to chemical weathering of clay minerals and decreased the base saturation^20–22^.

However, these reaction conditions are unrealistic in natural environment after acid or alkali spills. To predict the pH buffering capacity of acid- or alkali-spilled clay minerals, clay minerals’ site density and surface reaction constants should be determined. It has been known that two major pH buffering reactions of clay minerals are protonation or deprotonation reaction of edge sites, and proton exchange reaction of basal sites^23,24^. Previous studies have successfully identified the site density of surface functional groups in clay minerals and their surface reaction constants using surface complexation modeling^25,26^. Nevertheless, a few studies exist investigating alterations of surface properties of clay minerals at ambient temperature following intensive acid or alkali treatment, which is similar to acid or alkali spills, by using surface complexation modeling. In addition, clay minerals play a significant role in the pH buffering capacity of soils with low organic matter content resulting from acid or alkali spills; thus, knowledge of the changes in clay minerals’ properties after these spills is essential to interpret the pH buffering capacity of the soils.

The goals of this study were to investigate changes in the site density and surface reaction constants of the neutralized clay minerals after acid or alkali spills and evaluate their effect on the pH buffering capacity as an indicator of soil function. Kaolinite (KGa-1b, Georgia) and montmorillonite (SWy-3, Wyoming), which are typical 1:1 and 2:1 clay minerals, respectively, were selected in this study. Three different samples (untreated, acid-spilled, and alkali-spilled) were prepared by treating with deionized water, 5 M HCl, and 5 M NaOH, respectively, and they were neutralized, and their surface properties before and after acid or alkali spills were compared. The site density and surface acidity constants were determined by a potentiometric titration using surface complexation modeling, and the change in the pH buffering capacity resulting from acid or alkali spills was investigated.

## Results and Discussion

### XRD characterization of three different clay minerals

**Figure 1:**
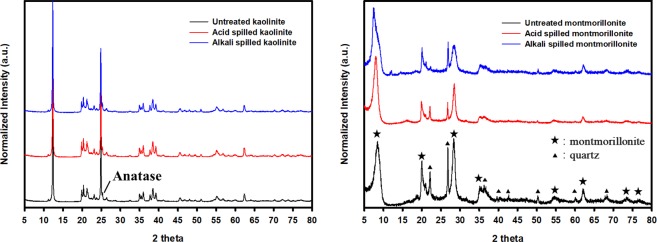
XRD patterns of three different (left) kaolinite and (right) montmorillonite samples. Black, red, and blue lines represent the XRD patterns of untreated, acid-spilled, and alkali-spilled clay minerals, respectively. Kaolinite and montmorillonite contained an anatase and a quartz as an impurity, respectively, which is consistent with the previous baseline study on the XRD patterns of KGa-1b kaolinite and Swy-2 montmorillonite^24^.

The XRD patterns of three different kaolinite and montmorillonite samples are shown in Fig. 1, and the results of quantitative analysis are summarized in Supplementary Table S1. The untreated kaolinite and montmorillonite were mainly composed of pure kaolinite (98%) and montmorillonite (79%), respectively. The untreated kaolinite contained an anatase (2%), and the untreated montmorillonite contained a quartz (13%) and cristobalite (8%). The results are consistent with the previous baseline study on the XRD patterns of KGa-1b kaolinite and Swy-2 montmorillonite^27^.

The XRD patterns and quantitative analysis showed that while acid or alkali spills had little effect on the crystalline structure and the mineral identity of kaolinite, they had some effects on those of montmorillonite. Newly formed constituents were not observed in both acid- or alkali-spilled kaolinite and montmorillonite. The proportions of kaolinite and anatase in both acid- and alkali-spilled kaolinite did not change as a result of each spill. In montmorillonite samples, however, acid spills did not significantly change the proportion of montmorillonite, while decreased that of quartz to 4% and increased that of cristobalite to 14%. Alkali spills increased the proportion of montmorillonite to 88%, while decreased those of quartz and cristobalite to 9 and 3%, respectively.

### Dissolution of major elements of clay minerals by acid or alkali spills

The concentrations of dissolved elements of kaolinite and montmorillonite as a result of acid or alkali spills are summarized in the Table 1. The dissolved Al and Si concentrations of kaolinite after acid spill were 2.67 mg-Al g^−1^ and 0.737 mg-Si g^−1^, respectively, and those after alkali spill were 21.8 mg-Al g^−1^ and 25.0 mg-Si g^−1^, respectively. Kaolinite was more susceptible to alkali spill than to acid spill. In the case of montmorillonite, while the dissolved Al and Si concentrations after acid spill were 9.09 mg-Al g^−1^ and 18.8 mg-Si g^−1^, respectively, those after alkali spill were 1.24 mg-Al g^−1^ and 103 mg-Si g^−1^, respectively. While more than five times of the octahedral cations such as Al, Fe and Mg were dissolved by acid spill than by alkali spill, the tetrahedral cation, Si, was dissolved five times more by alkali spill than by acid spill. The high dissolution of octahedral cations as a result of acid spill was probably because protons preferentially attack the isomorphic substituted elements in octahedral sheet during acid treatment^28^.

**Table 1:** Dissolution of the clay minerals’ constituent elements by acid or alkali spills measured by ICP-OES.

Clay minerals	Condition	Concentration of Dissolved Constituent Elements			
		Al	Si	Fe	Mg
**mg (g clay minerals)** ^**−1**^					
Kaolinite	Acid	2.67 ± 0.15	0.74 ± 0.04	0.25 ± 0.01	0.03 ± 0.00
	Alkali	21.8 ± 1.00	25.0 ± 2.3	0.48 ± 0.14	0.02 ± 0.00
Montmorillonite	Acid	9.09 ± 1.56	18.8 ± 6.2	11.3 ± 1.19	1.99 ± 0.34
	Alkali	1.24 ± 0.28	104 ± 11.5	2.61 ± 0.16	0.11 ± 0.06

The concentration of dissolved elements are the average values of triplicate experiments.

### Irreversible alteration on site density and surface acidity constants resulting from acid or alkali spills

**Figure 2:**
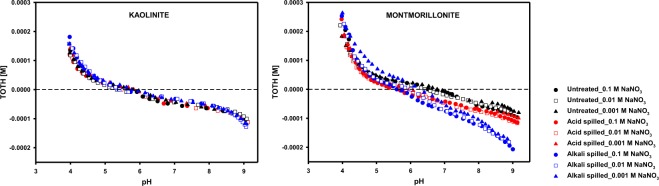
Titration curves of three different (left) kaolinite and (right) montmorillonite samples obtained from potentiometric titrations. The Y-axis is the molar concentration of the total added proton in the solution. The black, red, and blue symbols represent untreated, acid-spilled, and alkali-spilled kaolinite or montmorillonite, respectively, and the circle-, rectangle-, and triangle-shaped symbols represent a 0.1, 0.01, and 0.001 M NaNO_3_ condition, respectively. All experimental data were well fitted with predicted titration curves using SCM (see Supplementary Fig. S1).

The titration curves of three different kaolinite and montmorillonite samples under different NaNO_3_ concentration are shown in Fig. 2. As shown, acid or alkali spills have a greater effect on the titration curves of montmorillonite than those of kaolinite, and the background electrolytic concentration had a limited effect on the titration curves of both kaolinite and montmorillonite at pH values of 4–9. The reversibility test of three different kaolinite and montmorillonite samples under 0.01 M NaNO_3_ are shown in Supplementary Fig. S3. Although some hysteresis was observed, it was not pronounced. It could be because the factors such as CO_2_ input and dissolutions of clay minerals were controlled during the experiments^29^. The optimized values of the surface properties obtained from FITEQL 4.0 are summarized in Table 2.

**Table 2:** Optimized surface properties of three different kaolinite and montmorillonite samples using FITEQL.

	Kaolinite			Montmorillonite		
	Untreated	Acid-spilled	Alkali-spilled	Untreated	Acid-spilled	Alkali-spilled
**Surface reaction constants (pK)**						
$\equiv \,SO{H}_{2}^{+}\leftrightarrow \,\equiv SOH+{H}^{+}\,\,{({\rm{p}}{\rm{K}}{\rm{a}}}_{1})$	−4.55	−4.38	−4.72	−5.79	−5.93	−5.00
$\equiv \,SO{H}_{2}^{+}\leftrightarrow \,\equiv SO{H}^{-}+{H}^{+}\,\,{({\rm{p}}{\rm{K}}{\rm{a}}}_{2})$	5.49	5.94	6.34	7.32	5.42	6.78
$\equiv \,{X}^{-}\cdot N{a}^{+}+{H}^{+}\leftrightarrow \,\equiv {X}^{-}\cdot {H}^{+}+N{a}^{+}\,\,{({\rm{p}}{\rm{K}}}_{{\rm{H}}})$	−2.90^a^	−2.90^a^	−2.90^a^	1.51	0.72	1.85
**Functional group density**						
Edge site density (mmol kg^−1^)	35.8	40.6	38.6	84.8	85.3	253.2
Basal site density (mmol kg^−1^)	8.3	5.9	8.2	832.0	737.0	925.0

The Ka_1_, Ka_2_, edge site density and basal site density were fitting parameters and optimized. In montmorillonite samples, K_H_ was also included in fitting parameters. The detailed procedure of parameter optimization is summarized in the Supplementary Information.

^a^Not determined by optimization, but the average value of other research^55,68–70^.

Table 2 shows that an acid or alkali spill did not significantly change the edge and basal site density, which refers to variable charged sites on the mineral edges and permanent negatively charged sites on basal planes, respectively, of kaolinite (i.e. less than 5 mmol kg^−1^). An acid or alkali spill increased the edge site density of kaolinite from 35.8 to 40.6 and 38.6 mmol kg^−1^, respectively, and changed the basal site density of kaolinite from 8.3 to 5.9 and 8.2 mmol kg^−1^, respectively. In the case of montmorillonite, an acid or alkali spill significantly changed the edge and basal site density (i.e. greater than 100 mmol kg^−1^) except for the edge site density of the acid-spilled montmorillonite. An alkali spill greatly increased the edge site density from 84.8 to 253.2 mmol kg^−1^. In addition, an acid or alkali spill changed the basal site density from 832 to 737 and 925 mmol kg^−1^, respectively.

Regarding the surface acidity constants (i.e. pKa_1_ and pKa_2_), an acid or alkali spill did not cause meaningful changes in the kaolinite (i.e. less than a 0.5 pKa value) except the pKa_2_ of the alkali-spilled kaolinite increased from 5.49 to 6.34. In the case of montmorillonite, the pKa values greatly changed compared to those of the kaolinite (i.e. greater than a 0.5 pKa value) except for the pKa_1_ value of the acid-spilled montmorillonite. The pKa_1_ value of the alkali-spilled montmorillonite increased from −5.79 to −5.00, while the pKa_2_ value of the acid-spilled montmorillonite greatly decreased from 7.32 to 5.42 and that of the alkali-spilled montmorillonite decreased to 6.78. In addition, the proton exchange reaction constant of montmorillonite (pK_H_) changed from 1.51 to 0.72 and 1.85 as a result of an acid or alkali spill, respectively.

According to the Fig. 2, titration curves of the kaolinite and montmorillonite only slightly changed under the different concentrations of background electrolyte. In the case of kaolinite, this was probably because the edge site density was approximately five times higher than the basal site density, which was consistent with previous studies^24,30^. Thus, the effect of the proton exchange reaction of the kaolinite’s basal sites on the titration curve was relatively small in a range of pH 4–9 (see Supplementary Fig. S2). In contrast to the kaolinite, the basal site density of montmorillonite was approximately 5–10 times higher than its edge site density, which was consistent with previous research summarized in Bourg *et al*.^25^. However, the concentration of the electrolyte also had a slight effect on the titration curves of montmorillonite, and this might be because montmorillonite used in this study has a high affinity to Na^+^. Basal sites were saturated with Na^+^ at pH values of 4–9; thus, the proton exchange reaction could be negligible within this pH range (see Supplementary Fig. S2). This indicated that the pH buffering of both kaolinite and montmorillonite was largely determined by the edge site reactions rather than those of the basal sites at pH 4–9. Thus, it is reasonable that the larger change in the titration curves of the montmorillonite compared to that of the kaolinite resulting from acid or alkali spills was probably because of the greater alterations on the edge sites’ properties in the montmorillonite.

### Structural modification due to acid or alkali spills

**Figure 3:**
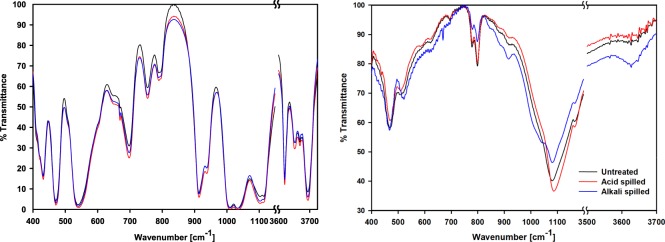
FT-IR spectra of (left) kaolinite and (right) montmorillonite. Black, red, and blue lines represent untreated, acid-spilled, and alkali-spilled kaolinite or montmorillonite.

FT-IR spectra of the three different kaolinite and montmorillonite samples are shown in Fig. 3; the band assignments of the Clay Minerals Society’s source clay (KGa-1b kaolinite, Swy-2 montmorillonite) were used in this study^31,32^. All absorption bands, particularly the Si-O of the tetrahedral sheet at 400–1100 cm^−1^, Si-O-Si at 472 cm^−1^, Al-O-Si of the octahedral sheet at 541 cm^−1^, and the OH hydroxyl groups at 915, 938, and 3600–3700 cm^−1^, of the untreated kaolinite were observed and the positions of the bands were nearly the same as the reference. However, the untreated montmorillonite (Swy-3) used in this study had a similar but slightly different shape and position of bands compared to the Swy-2 montmorillonite in the reference. The absorption band assigned for Al-O-Si, found in Swy-2 at 524 cm^−1^, was observed near 512 cm^−1^, and the band assigned for the Si-O of the tetrahedral sheet, found in Swy-2 montmorillonite at 1041 cm^−1^, was not observed in the untreated montmorillonite. Instead, the strong band at 1080 cm^−1^ was observed, and it might be because the untreated montmorillonite contained some quartz and cristobalite whose bands assigned to the Si-O were observed near 1080 cm^−1^ ^31^. It is supported by the FT-IR spectra observed at 778 and 798 cm^−1^, which are assigned to the Si-O of quartz and cristobalite, respectively. These results are consistent with the result of XRD analysis (Fig. 1 and Table S1) and the FT-IR baseline study. The other bands were similar to those of the Swy-2 montmorillonite. Absorption bands at 842, 885, and 917 cm^−1^ were assigned to octahedral sites where isomorphic substitution occurred, and the band at 3627 cm^−1^ was assigned to the hydroxyl groups of octahedral cations, particularly Al^3+^ ^33^.

Acid or alkali spills did not significantly change the FT-IR spectra of the kaolinite. All the absorption bands were in the same position and the intensity of the bands was minimally altered. This indicated that limited structural alteration occurred. This is consistent with little changes in XRD patterns and quantitative analysis of kaolinite after an acid or alkali spill (Fig. 1 and Table S1). It is also supported by the results of the titration experiment in which surface reaction constants and site density did not appreciably change as a result of an acid or alkali spill (Table 2). In addition, previous studies revealed the similar result that kaolinite, which is one of the non-swelling minerals, is the most resistant clay mineral to acid or alkali attacks^8,34^.

The acid- or alkali-spilled montmorillonite showed different FT-IR spectra compared to those of the untreated montmorillonite (Fig. 3). As a result of acid spill, the intensity of the bands assigned to the isomorphically substituted octahedral cation sites (i.e., Al-(Mg, Fe, Al)-OH) decreased. This result was probably because of the great amount of dissolution of Fe and Mg in montmorillonite after acid spill (Table 1). It is also supported by the XRD quantitative analysis that the proportion of montmorillonite did not change by acid spills. It implied that the decrease in the basal site density of the acid-spilled montmorillonite was due to the dissolution of isomorphically substituted octahedral cation sites. In addition, acid spill decreased the intensity of the band for the Si-O-Si sites of montmorillonite, while the Si-O band at 1080 cm^−1^ was shifted to 1090 cm^−1^ at which the Si-O band of cristobalite was assigned^31^. Together with the results of the XRD quantitative analysis (Table S1), the FT-IR spectra indicated that acid spills dissolved Si from montmorillonite and cristobalite was formed. Madejová also observed the similar trend in smectite that the amorphous silica was newly formed after acid treatment^32^.

In contrast to acid spill, alkali spill increased the intensity of the band assigned to the isomorphically substituted octahedral cation sites of montmorillonite, which corresponds with the optimized basal site density in Table 2. Alkali spill increased basal site density by about 10% (Table 2), which matched with the increasing ratio of montmorillonite by alkali spills (Table S1); Thus, the possible reason for the increase can be ascribed to the enrichment of montmorillonite by alkali spill. Regarding the Si environment in montmorillonite, alkali spill decreased the intensity of Si-O bands of quartz and cristobalite at 778, and 798 cm^−1^, respectively, which is consistent with the XRD results of alkali-spilled montmorillonite. The absorption band assigned to the Si-O of the tetrahedral sheet, which was not observed in the untreated or acid-spilled montmorillonite, appeared in the alkali-spilled montmorillonite at 1041 cm^−1^. In addition, the intensity of the band assigned to hydroxyl group at 3627 cm^−1^ increased by alkali spills, which corresponds to the increase in the edge site density of alkali-spilled montmorillonite from titration experiment (Table 2). According to Table 2, the alkali-spilled montmorillonite had different Ka values with those of the untreated montmorillonite. The results of FT-IR analysis and optimized Ka values indicate that alkali spills increased the proportion of montmorillonite by dissolving quartz and cristobalite, and also changed the surface functional groups’ properties by forming new AlOH and SiOH in montmorillonite.

FT-IR spectra demonstrated that montmorillonite was more vulnerable to acid or alkali spills compared to kaolinite, and had good agreement with the optimized site density (Table 2). This was probably because H^+^ and OH^−^ could attack not only the edges but also the swollen interlayer of the montmorillonite^8,34^. The results indicated that the extent of change in the surface properties resulting from an acid or alkali spill varied with the type of clay mineral, and especially, an expandability should be carefully considered.

### Interpretation of the change in Ka_2_ using first principle molecular dynamics results

Experimentally derived Ka represented the average Ka of the reactive edge sites in the clay minerals. It is difficult to directly compare the Ka derived from the titration experiment to those calculated from first principle molecular dynamics (FPMD)^35,36^. In addition, the interpretation of pKa_1_, generally less than 0, optimized from the titration experiment was inherently limited because the titration data at the pH near pKa_1_ were meaningless because of the dissolution of the clay minerals. However, a previous study showed that the experimental Ka_2_, optimized by assuming one edge site with a constant capacitance model, well matched the Ka calculated from FPMD^37^; thus, it is possible to interpret pKa_2_ in terms of the theoretically calculated pKa. Liu *et al*. compared the optimized Ka values of kaolinite and montmorillonite from various titration experiments with the theoretical values calculated based on FPMD; these values are summarized in Supplementary Table S2^37,38^. The pKa values of three different kaolinite and montmorillonite samples optimized from this study coincided well with the calculated and experimentally fitted values from other references. This indicated that the edge sites detected from the titration experiments represented surface functional groups as summarized in Supplementary Table S2.

The pKa_2_ of the untreated and acid-spilled kaolinite (5.49 and 5.94) was similar to calculated pKa of AlOH sites of kaolinite (5.7), while that of the alkali-spilled kaolinite (6.34) was near the average value of calculated pKa of AlOH and SiOH sites of kaolinite (6.3). However, considering little changes in kaolinite samples’ titration curves, XRD, and FT-IR, the differences in pKa values were just theoretically generated during the optimization process. In case of montmorillonite, the pKa_2_ of the untreated montmorillonite (7.32) was similar with the average value of calculated pKa of SiOH and AlOH sites of montmorillonite (7.87), while that of the acid-spilled montmorillonite (5.42) was near the calculated pKa of AlOH sites of montmorillonite (approximately 5.5). It indicated that acid spills decreased SiOH sites of montmorillonite and this is consistent with FT-IR spectra results that acid spills dissolved Si from the untreated montmorillonite and formed the cristobalite. Because theoretical pKa of edge sites was calculated from pure clay minerals, the formation of cristobalite would make pKa of the acid-spilled montmorillonite further different from the untreated montmorillonite. Also, decrease in substituted octahedral sites of the acid-spilled montmorillonite might affect pKa_2_, because isomorphic substitutions increased the adjacent edge sites’ pKa^37,38^. In the case of the alkali-spilled montmorillonite, pKa_2_ (6.78) was between that of the untreated and acid-spilled montmorillonite. Both SiOH and AlOH increased after alkali spills (Fig. 3); thus pKa_2_ changed less than the acid-spilled montmorillonite. These results indicated that changes in pKa_2_ by acid or alkali spills was primarily because of dissolution or formation of surface functional groups, which have different theoretical pKa values.

### Effect of acid or alkali spills on pH buffering capacity

**Figure 4:**
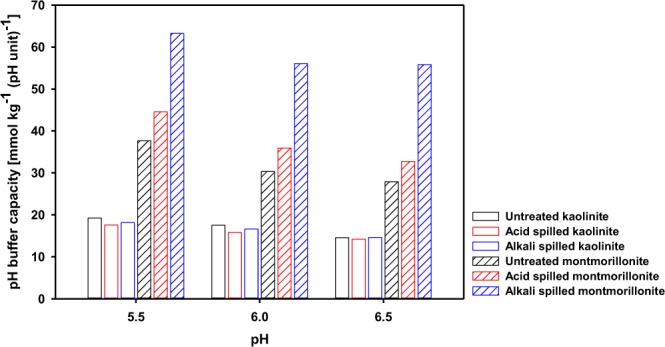
pH buffering capacity of three different kaolinite and montmorillonite samples at pH 5.5, 6.0, and 6.5. Open and cross-hatched bars represent kaolinite and montmorillonite, respectively, and black, red, and blue bars represent untreated, acid-spilled, and alkali-spilled kaolinite or montmorillonite, respectively.

The pH buffering capacity was the reciprocal of the slope of the titration curve, and it was calculated from the following Equation (1):

1 $${\rm{pH}}\,{\rm{buffering}}\,{\rm{capacity}}\,{\rm{at}}\,{\rm{pH}}{\rm{x}}=-({{\rm{TOTH}}}_{{\rm{pH}}{\rm{x}}+{\rm{0.1}}}-{{\rm{TOTH}}}_{{\rm{pH}}{\rm{x}}})/{\rm{0.1}}$$

where, TOTH_pH x_ (mol L^−1^) is the total amount of proton added to the suspension of clay minerals during the titration until the suspension pH reaches a value of x. Because the initial pH of the suspension of all the clay minerals ranged from 5.5 to 6.5, the pH buffering capacity at pH 5.5, 6.0 and 6.5 was calculated from the titration curves predicted by using surface complexation modeling under 0.001 M NaNO_3_ condition (Fig. 4).

Acid or alkali spills slightly decreased the pH buffering capacity of the kaolinite at pH 5.5, 6.0, and 6.5 from 19.2, 17.6, and 14.6 mmol kg^−1^ to 17.6 or 18.2, 15.8 or 16.6, and 14.2 or 14.6 mmol kg^−1^, respectively. In the case of montmorillonite, these spills increased the pH buffering capacity at pH 5.5, 6.0 and 6.5 from 37.7, 30.3 and 27.9 mmol kg^−1^ to 44.5 or 63.2, 35.9 or 56.0, and 32.7 or 55.8 mmol kg^−1^, respectively. Because the surface reaction constants and site density determined the pH buffering capacity, the pH buffering capacity of montmorillonite, of which the surface properties were greatly altered by the acid or alkali spills compared to those of kaolinite, was more affected by these spills than kaolinite.

Acid or alkali spills do not result in meaningful changes in the pH buffering capacity of kaolinite, but, unexpectedly, increased the pH buffering capacity of montmorillonite. It indicated that acid or alkali spills do not have an adverse effect on the pH buffering capacity of clay minerals, which is the indicator of soil functions, after neutralization. However, in reality, soils contain not only clay minerals, but also organic matter; thus, the pH buffering capacity of acid- or alkali-spilled soils could change in a different manner from that of a single clay mineral alone. Organic matter is known as the most important component determining pH buffering^23^, and it may have a pH buffering capacity 300 times higher than that of kaolinite^39^. Because acid or alkali spills would decrease organic matter contents via desorption and dissolution^14–16^, these spills might cause a decrease in the pH buffering capacity of acid- or alkali-spilled soils despite an increase in the pH buffering capacity of single clay minerals. Nevertheless, the results of the pH buffering capacity of clay minerals in this study clearly show that the pH buffering capacity was not deteriorated, but rather increased in montmorillonite after acid or alkali spills.

## Conclusions

In this study, we investigated the change in surface properties of clay minerals due to acid or alkali spills and its effect on the pH buffering capacity as an indicator of soil functions. Surface complexation modeling indicated that acid or alkali spills did not significantly change the site density of surface functional groups and their surface acidity constants in kaolinite. In contrast, acid or alkali spills greatly changed the site density and surface acidity constants of montmorillonite. Acid spills dissolved isomorphically substituted octahedral cations and SiOH sites in montmorillonite. In addition, alkali spills dissolved the quartz and cristobalite and edge sites such as AlOH and SiOH sites were newly formed in montmorillonite. Regarding the pH buffering capacity of clay minerals, acid or alkali spills did not have an adverse effect on it, but rather increased in montmorillonite. Soil generally contains organic matter, which plays a significant role in pH buffering, and thus further research with field soil is needed to delineate the effect of acid or alkali spills.

## Materials and Methods

### Preparation and characterization of clay minerals

KGa-1b kaolinite (Georgia) and SWy-3 montmorillonite (Wyoming) were purchased from the Clay Minerals Society’s Source Clays Repository. All chemicals used in this study were of extra pure or reagent grade. The clay minerals were prepared following a similar procedure to the best practices for analyzing surface properties of montmorillonite reviewed by Duc *et al*.^40–43^. Thirty grams of kaolinite and montmorillonite was dispersed in 1 L of deionized water with a specific resistance of 18.2 MΩ m (Milipore, Bedford, Ma, USA) for 4 h. Prior to the size separation, the pH value of the kaolinite suspension was adjusted to 9.5 by adding NaOH (98%, Daejung, Korea) to facilitate a dispersion. The <2-μm fraction of each clay mineral was collected via centrifugation (119 g, 5 min). This fraction was washed with 1 M NaNO_3_ (99%, Daejung, Korea) and HNO_3_ (60%, Daejung, Korea) solution at pH 3 and the supernatant was decanted after centrifugation. This decarbonating procedure was repeated until the supernatant pH reached 3^41,42,44–46^. The clay minerals were then collected via centrifugation and washed three times with 1 M NaNO_3_ solution to change the clays to a Na^+^ form. Although three washing cycles with NaNO_3_ might not be enough to make clay minerals homoionic^47^, this pretreatment was chosen to allow for closer comparisons to previous studies, which determined clay minerals’ Ka and site density through surface complexation modeling^30,44,48^. Excess Na^+^ was removed by washing five times with deionized water, and the final pH was in the range from 6 to 7. The collected clay minerals were freeze-dried, and the clay minerals are referred to as untreated clay minerals.

The CEC of untreated kaolinite and montmorillonite, measured ammonium ion through the ammonium acetate method^49^, was 44 and 832 mmol kg^−1^, respectively. The specific surface areas of the untreated kaolinite and montmorillonite, obtained using N_2_- Brunauer–Emmett–Teller (BET) analysis (ASAP2020, Micromeritics, USA) through adsorption (32 points) and desorption (23 points) isotherms (see in Supplementary Fig. S4)^50^, were 12.10 and 33.75 m^2^ g^−1^, respectively. These values were used as parameters for the surface complexation modeling^30,51–54^.

### Acid or alkali spill and neutralization

HCl (35%, Daejung, Korea) and NaOH were selected as the strong acid and alkali, respectively, based on their frequency of chemical accidents and amount of use^1,3^. One gram of the untreated kaolinite or montmorillonite was placed in a 50-mL conical tube and 45 mL of 5 M HCl or NaOH was added to simulate an extreme acid or alkali spill situation. Whole reactions were conducted in a rotating shaker at 25 °C and 40 rpm for two days. The suspension was centrifuged and the supernatant solutions were filtered through a 0.22-μm filter (Whatman, UK). After acid or alkali spill experiments, the treated solutions were analyzed by using inductively coupled plasma optical emission spectrometry (ICP-OES, iCAP 7400, Thermo Fisher Scientific, USA) to determine the dissolution of the major structural constituents of kaolinite and montmorillonite such as Al, Si, Fe, and Mg.

Separated kaolinite or montmorillonite were washed with deionized water five times to remove excess salts and dissolved ions. A neutralization process was needed because excess H^+^ and OH^−^ remaining after washing and decanting could affect the titration experiment. Thus, the washed kaolinite or montmorillonite were neutralized by adding HNO_3_ or NaOH until the supernatant pH reached a neutral pH range. The suspensions were centrifuged and decanted and then residual clay minerals were washed three times with 1 M NaNO_3_ to make them homoionic. These clay minerals were washed five times with deionized water, and then freeze-dried. The concentrations of Al and Si in the suspensions were measured by ICP-OES to check the remaining ions’ concentration, and they were below one mg kg^−1^. The whole experiments of acid or alkali spills and neutralization were carried out in triplicates. The XRD analysis was conducted to analyze the mineral identity of the acid- or alkali-spilled clay minerals. The CEC of acid- or alkali-spilled kaolinite was 4.7 cmol kg^−1^, while that of acid- or alkali-spilled montmorillonite was 737 and 925 mmol kg^−1^, respectively. The specific surface area of the acid- or alkali-spilled kaolinite was 13.60 and 14.14 m^2^ g^−1^, respectively, while that of the acid- or alkali-spilled montmorillonite was 34.88 and 19.32 m^2^ g^−1^, respectively (see in Supplementary Fig. S4).

### Potentiometric titration

Potentiometric titration has been used to quantify the density of active reaction sites of clay minerals and determine the surface acidity constants (Ka) of those sites^25,26,43,44,46,54–61^. The protonation behaviors of three different kaolinite or montmorillonite (untreated, acid-spilled, and alkali-spilled) were investigated using an automatic potentiometric titrator (G10S, Mettler-Toledo, Switzerland). Duc *et al*. recommended the best experimental conditions for the titrations to analyze the acid-base properties of montmorillonite^42,43,62^, and we adapted the procedure with some modifications as follows.

All titration experiments were performed within a pH range of 4–9 at 25 ± 1 °C under an N_2_-purging condition. Deionized water was boiled to remove CO_2_ before making clay suspensions and the base titrant. Kaolinite (0.1 g) or montmorillonite (0.05 g) was dispersed in 50 mL of background electrolyte (NaNO_3_) solution, and the suspensions were purged with N_2_ for 1 h to exclude CO_2._ The suspensions were continuously stirred and purged with N_2_ during each titration. Titrations of each clay suspension were conducted at three different concentrations of NaNO_3_ (0.1, 0.01, and 0.001 M) to investigate the effect of the background electrolytic concentration. Titrant solutions (0.1 M HNO_3_ or NaOH), also purged with N_2_, were added to the suspensions as 5–10-μL increments every 5 min, and the pH was automatically recorded. Previous research has demonstrated that a short time interval between successive increments of titrant, generally less than 10 min, is reasonable to minimize mineral dissolution^43,46,56,59,63^. Drifts in the measured potential were less than 1 mV min^−1^ in all titration experiments. Two independent titrations of each clay suspension, from initial pH to pH 4 and from initial pH to pH 9, were conducted and combined to obtain one titration curve ranging from pH 4 to 9. This pH range was selected because it is known that the dissolution of kaolinite and montmorillonite is negligible in this pH range^44,57,58^. In addition, to check the reversibility of titration experiments, titrations from pH 4 to pH 9 were conducted at 0.01 M NaNO_3_ condition.

### X-ray diffraction measurement

X-ray diffraction (XRD) pattern of the samples of kaolinite and montmorillonite was measured with a X-ray diffractometer (D8 ADVANCE with DAVINCI, Bruker, German) using Cu Kα radiation with a λ of 1.5418 Å and a Lynxeye-XE detector operating at 40 kV and 40 mA at a scan rate of 2.4° min^−1^ from 5° to 80°. The TOPAS Rietveld analysis of XRD patterns was used to quantify the content of mineral components of kaolinite samples. Since it is not precise and difficult to quantify montmorillonite by Rietveld analysis due to the stacking disorder^64,65^, the fraction of constituents was analyzed semi-quantitatively by comparing the integrated intensities of the diffraction peaks of each constituents^66^.

### Fourier-transform infrared spectroscopy

Fourier-transform infrared (FT-IR) spectroscopy was used to investigate the mineralogical and chemical structure and active surface sites of clay minerals for surface acidity^31,32^. Clay samples were dried at 110 °C overnight to minimize water adsorption. KBr pellets were prepared by mixing kaolinite (1.5 mg) or montmorillonite (1 mg) with 240 mg of KBr powder at a pressure of 10 tons. The FT-IR spectra of the pellets with kaolinite or montmorillonite were recorded using an FT-IR spectrometer (Nicolet 6700, Thermo Scientific, USA) with 32 scans at a resolution of 4 cm^−1^ within the range of 400–4,000 cm^−1^.

### Surface complexation modeling

A constant capacitance model with permanent negatively charged sites on basal plane and variable charged sites on the mineral edges was chosen to model the surface properties of the kaolinite and montmorillonite^30,37,44^. The former was referred to as basal sites, while the latter as edge sites. A proton exchange reaction occurred on the basal sites of the clay minerals, while a protonation or deprotonation reaction occurred on the edge sites of them. The least square fitting program FITEQL 4.0 was used to optimize the site densities of the kaolinite and montmorillonite and their surface reaction constants from the titration experiment^67^. The CEC of the kaolinite and montmorillonite was used as the total site density of the kaolinite and the basal site density of the montmorillonite, respectively^25,30,44^. Surface properties were optimized from titration data at three different concentrations of background electrolyte (0.1, 0.01, and 0.001 M NaNO_3_). FITEQL failed to converge without fixing the equilibrium constant of the kaolinite’s proton exchange reaction probably because the titration curves of the kaolinite could be well described without considering the basal sites’ proton exchange reaction. Thus, pK_H_ of the kaolinite’s proton exchange reaction constant was assumed to be −2.9 (Table 2), which is an averaged value from references^55,68–70^. Detailed descriptions of the surface complexation modeling used in this study and the optimization process are explained in the Supplementary Information.

## Supplementary information

Supplementary Information

## Data Availability

The datasets generated during and/or analyzed during the current study are available from the corresponding author on reasonable request.
