# Supplementary material for: Change in the site density and surface acidity of clay minerals by acid or alkali spills and its effect on pH buffering capacity
Source: Sci Rep. 2019 Jul 8;9:9878. doi: 10.1038/s41598-019-46175-y (PMC6614462; doi:10.1038/s41598-019-46175-y)
Supplement: Supplementary file 1 — Supplementary Information [file 41598_2019_46175_MOESM1_ESM.docx]

**Supplementary Information**

**Change in the site density and surface acidity of clay minerals by acid or alkali spills and its effect on pH buffering capacity**

**Inhyeong Jeon^1^, Kyoungphile Nam^1,^***

^1^Department of Civil and Environmental Engineering,

Seoul National University, Seoul 08826, Republic of Korea

Address: 35-307, Seoul National University, 1 Gwanak-ro, Gwanak-gu

E-mail: kpnam@snu.ac.kr

Phone: +82 2 880 1448

Fax: +82 2 873 2684

**S1. Description of surface complexation modeling**

Surface complexation modeling (SCM) has been developed to describe and predict metal cation and anion adsorption reactions for a wide range of sorbents, including oxide minerals and phyllosilicate clay minerals ^1-7^. Previous studies have showed that SCM could successfully explain proton and metal adsorption onto kaolinite or montmorillonite ^8,9^.

Kaolinite and montmorillonite are generally regarded as having two types of surface sites, permanent negatively charged sites on basal plane ($\equiv X^{-}$) and variable charged sites on the mineral edges ($\equiv SOH$). The former, referred to as a basal site, have the permanently negative charge because of isomorphic substitution of clay minerals, and account for the cation exchange reaction, while the latter, referred to as an edge site, contribute to proton or metal binding at the clay mineral edges. Gu et al and references therein showed that a constant capacitance model with two sites, including one basal site and one amphoteric edge site, could successfully describe the adsorption properties of kaolinite and montmorillonite ^10,11^. In addition, the surface acidity constants (pKa) of kaolinite and montmorillonite obtained from using this model had good agreement with those calculated with first principle molecular dynamics (FPMD) ^12,13^. In some studies, the edge sites were divided into aluminol and silanol sites ^14-16^, but they are difficult to distinguish only by titration experiments because the two sites have very close Ka values at a normal pH (3<pH<9). Thus, one amphoteric edge site, representing the average of the reactive surface functional groups, was assumed, and the constant capacitance model was used to model the electrostatic properties of the clay minerals in this study.

**S2. Optimization using FITEQL to obtain the surface reaction constants and site density of the kaolinite and montmorillonite**

A total of six parameters (surface acidity constants (Ka_1_, Ka_2_), proton exchange reaction constant (K_H_), edge and basal site density, and capacitance (κ)) were set as adjustable parameters for optimization. In the case of kaolinite, the cation exchange capacity (CEC) was used as the total site density, and the pK_H_ of the proton exchange reaction was assumed to be -2.9. Titration data under 0.1 and 0.01 M NaNO_3_ conditions were used to optimize Ka_1_, Ka_2_, and the edge site density because the proton exchange reaction only slightly affected the titration curves under these conditions. The basal site density was calculated from the difference between the total site density and the averaged edge site density. Finally, all titration data were used to check the convergence with fixing all the averaged surface properties. This successive step was continued until a goodness-of-fit, indicated by the WSOS/DF value of the FITEQL, was between 0.1 and 20 ^17^. In the case of montmorillonite, the CEC was used as the basal site density. Titration data under 0.1 and 0.01 M NaNO_3_ conditions were used to optimize the Ka_1_, Ka_2_, and edge site density just as for kaolinite. In addition, titration data under a 0.001 M NaNO_3_ condition was used to optimize Ka_1_, Ka_2_, edge site density, and K_H_. All titration data were used to check the convergence with fixing all averaged surface properties, and this successive step was also continued until the convergence was achieved. In this study, κ was treated as an adjustable parameter for convergence because it cannot be experimentally determined ^18^. The κ of untreated, acid-spilled, and alkali-spilled kaolinite was 2.4 Farad m^-2^, and that of untreated, acid-spilled, and alkali-spilled montmorillonite was 2.8, 1.9, and 8.0 Farad m^-2^, respectively.

**Figure S1.** Titration curves of three different (up) kaolinite and (down) montmorillonite samples under three different concentrations of NaNO_3_. Symbols are experimental data and lines are fitted data calculated using SCM. Black, red, and blue symbols or lines are those under a 0.001, 0.01, and 0.1 M NaNO_3_ condition, respectively.

**Figure S2.** Predicted surface speciation diagrams of three different (left) kaolinite and (right) montmorillonite samples at three concentrations of NaNO_3_. Blue lines represent the edge sites’ species (i.e. $\equiv{SOH}_{2}^{+}$, $\equiv SOH$ and $\equiv{SO}^{-}$) and red lines represent the basal sites’ species (i.e. $\equiv X^{-}\cdot{Na}^{+}$ and $\equiv X^{-}\cdot H^{+}$).

Figure S3. Reversibility test of three different (left) kaolinite and (right) montmorillonite samples at 0.01 M NaNO_3_. Closed circles represent one titration from initial pH to pH 4 and from initial pH to pH 9, and opened circles represent the other titration from pH 4 to pH 9. Black, red, and blue symbols represent the titration of untreated, acid-spilled, and alkali-spilled clay samples, respectively.

Figure S4. N_2_ adsorption and desorption isotherms of three different (left) kaolinite and (right) montmorillonite samples. Circle and triangle symbols represent adsorption and desorption isotherms, respectively, and black, red, and blue symbols represent those of untreated, acid-spilled, and alkali-spilled clay samples, respectively.

**Table S1**

Results of XRD quantitative analysis of three different montmorillonite samples.

|  | Component | | |
| --- | --- | --- | --- |
|  | Montmorillonite | Quartz (SiO_2_) | Cristobalite (SiO_2_) |
|  |  | Content (%) |  |
| Untreated montmorillonite | 80 | 12 | 8 |
| Acid-spilled montmorillonite | 82 | 4 | 14 |
| Alkali-spilled montmorillonite | 88 | 9 | 3 |

In the case of three different kaolinite samples, they contained the same proportions of kaolinite (98%) and anatase (2%).

**Table S2**

pKa values calculated based on FPMD and optimized from titration using one-edge site modeling, as summarized in Liu et al ^12,13^.

|  | Surface | Surface edge sites | Kaolinite | Montmorillonite |
| --- | --- | --- | --- | --- |
| **Calculated based on FPMD ^a^** | 010 | Si(OH) | 6.9 | 7.0 |
|  |  | Al(OH2)(OH2) / Al(OH2)(OH) | 0.2 / 5.7 | 3.1 / 8.3 |
|  |  | Al(OH2)(OH2) / Al(OH2)(OH) ^b^ | - | 4.9 / 8.5 |
|  | 110 | Si(OH) | - | 8.3 |
|  |  | AlSiOH | - | 1.7 |
|  |  | Al(OH2) | - | 5.5 |
|  |  | MgSiOH | - | 4.2 |
|  |  |  |  |  |
| **Optimized from titration** |  | SOH_2_^+^ / SOH | -4.63 to -3.24 / 6.16–9.18 | -6.04 to -2.97 / 5.26–8.5 |

^a^ pKa values between 0 and 10 from Liu et al ^12,13^.

^b^ One tetrahedral Si is substituted to Al.

**References**

1 Davis, J. A. & Kent, D. Surface complexation modeling in aqueous geochemistry. *Reviews in Mineralogy and Geochemistry* **23**, 177-260 (1990).

2 Dzombak, D. A. *Surface complexation modeling: hydrous ferric oxide* (John Wiley & Sons, 1990).

3 Goldberg, S. *Advances in agronomy* Vol. 47 233-329 (Elsevier, 1992).

4 Chen, M. A. & Kocar, B. D. Radium sorption to iron (hydr)oxides, pyrite, and montmorillonite: implications for mobility. *Environmental Science and Technology* **52**, 4023-4030, doi:10.1021/acs.est.7b05443 (2018).

5 Xie, Y., Helvenston, E. M., Shuller-Nickles, L. C. & Powell, B. A. Surface complexation modeling of Eu(III) and U(VI) interactions with graphene oxide. *Environmental Science and Technology* **50**, 1821-1827, doi:10.1021/acs.est.5b05307 (2016).

6 Komarek, M., Koretsky, C. M., Stephen, K. J., Alessi, D. S. & Chrastny, V. Competitive Adsorption of Cd(II), Cr(VI), and Pb(II) onto nanomaghemite: A Spectroscopic and Modeling Approach. *Environmental Science and Technology* **49**, 12851-12859, doi:10.1021/acs.est.5b03063 (2015).

7 Marcussen, H., Holm, P. E., Strobel, B. W. & Hansen, H. C. B. Nickel sorption to goethite and montmorillonite in presence of citrate. *Environmental Science and technology* **43**, 1122-1127 (2009).

8 Bourg, I. C., Sposito, G. & Bourg, A. C. Modeling the acid-base surface chemistry of montmorillonite. *Journal of Colloid and Interface Science* **312**, 297-310, doi:10.1016/j.jcis.2007.03.062 (2007).

9 Tournassat, C., Davis, J. A., Chiaberge, C., Grangeon, S. & Bourg, I. C. Modeling the acid-base properties of montmorillonite edge surfaces. *Environmental Science and Technology* **50**, 13436-13445, doi:10.1021/acs.est.6b04677 (2016).

10 Gu, X. & Evans, L. J. Surface complexation modelling of Cd(II), Cu(II), Ni(II), Pb(II) and Zn(II) adsorption onto kaolinite. *Geochimica et Cosmochimica Acta* **72**, 267-276, doi:10.1016/j.gca.2007.09.032 (2008).

11 Gu, X., Evans, L. J. & Barabash, S. J. Modeling the adsorption of Cd (II), Cu (II), Ni (II), Pb (II) and Zn (II) onto montmorillonite. *Geochimica et Cosmochimica Acta* **74**, 5718-5728, doi:10.1016/j.gca.2010.07.016 (2010).

12 Liu, X. *et al.* Acidity of edge surface sites of montmorillonite and kaolinite. *Geochimica et Cosmochimica Acta* **117**, 180-190, doi:10.1016/j.gca.2013.04.008 (2013).

13 Liu, X., Cheng, J., Sprik, M., Lu, X. & Wang, R. Surface acidity of 2:1-type dioctahedral clay minerals from first principles molecular dynamics simulations. *Geochimica et Cosmochimica Acta* **140**, 410-417, doi:10.1016/j.gca.2014.05.044 (2014).

14 Zachara, J. & Smith, S. Edge complexation reactions of cadmium on specimen and soil-derived smectite. *Soil Science Society of America Journal* **58**, 762-769 (1994).

15 Brady, P. V., Cygan, R. T. & Nagy, K. L. Molecular controls on kaolinite surface charge. *Journal of Colloid and Interface Science* **183**, 356-364 (1996).

16 Tertre, E., Castet, S., Berger, G., Loubet, M. & Giffaut, E. Surface chemistry of kaolinite and Na-montmorillonite in aqueous electrolyte solutions at 25 and 60°C: Experimental and modeling study. *Geochimica et Cosmochimica Acta* **70**, 4579-4599, doi:10.1016/j.gca.2006.07.017 (2006).

17 Herbelin, A. L. & Westall, J. C. FITEQL: A computer program for determination of chemical equilibrium constants from experimental data. *Version* **4**, 99-01 (1999).

18 Motta, M. M. & Miranda, C. Molybdate adsorption on kaolinite, montmorillonite, and illite: Constant capacitance modeling. *Soil Science Society of America Journal* **53**, 380-385 (1989).
